# Analysis of Human Papillomavirus (HPV) and Polyomaviruses (HPyVs) in Adenoid Cystic Carcinoma (AdCC) of the Head and Neck Region Reveals Three HPV-Positive Cases with Adenoid Cystic-like Features

**DOI:** 10.3390/v14051040

**Published:** 2022-05-13

**Authors:** Mark Zupancic, Stefan Holzhauser, Liquin Cheng, Torbjörn Ramqvist, Juan Du, Signe Friesland, Anders Näsman, Tina Dalianis

**Affiliations:** 1 Department of Oncology-Pathology, Karolinska Institutet, 17164 Stockholm, Sweden; mark.zupancic@ki.se (M.Z.); stefan.holzhauser@ki.se (S.H.); torbjorn.ramqvist@ki.se (T.R.); signe.friesland@ki.se (S.F.); anders.nasman@ki.se (A.N.); 2 Department of Head-, Neck-, Lung- and Skin Cancer, Theme Cancer, Karolinska University Hospital, 17164 Stockholm, Sweden; 3 Department of Microbiology, Tumour Biology and Cell Biology, Karolinska Institutet, 17177 Stockholm, Sweden; liquin.cheng@ki.se (L.C.); juan.du@ki.se (J.D.); 4 Department of Clinical Pathology, Karolinska University Hospital, 17176 Stockholm, Sweden

**Keywords:** adenoid cystic carcinoma, basaloid carcinoma, histopathology, HPV-related multiphenotypic sinonasal carcinoma, human papillomavirus, human papillomavirus carcinoma with adenoid cystic-like features, human polyomavirus, molecular pathology, salivary gland malignancies, pathology of tumours, virology

## Abstract

An aetiological role of human papillomavirus (HPV) and/or human polyomaviruses (HPyVs) has been proposed in adenoid cystic carcinoma (AdCC). Moreover, HPV-related multiphenotypic carcinoma (HMSC) was recently introduced as an emerging entity of the sinonasal region. Here, we primarily want to study the role of HPV/HPyV in a large AdCC cohort and, secondly, possibly identify and characterize HMSC. Tumour DNA from 68 patients initially diagnosed with AdCC between 2000 and 2012 was, therefore, tested for 27 HPV types and 10 HPyVs. HPV DNA-positive samples were micromorphologically re-evaluated, further stained for p16^INK4a^, S100, p63 and CD117 and tested for the presence of the MYB-NFIB fusion transcript. Notably, no samples were HPyV-positive, while one sinonasal and two tonsillar carcinomas were HPV- and p16-positive. After re-evaluating the micromorphology, immunohistochemistry and presence of fusion transcripts, all tumours had the same appearance and fitted within the diagnosis of HMSC, but in all these three cases, the morphology of the HMSC and basaloid squamous cell carcinoma was overlapping. We conclude that HPV and HPyV have no major role in AdCC. However, based on our data, we also suggest that HMSC should be considered as a basaloid variant of squamous cell carcinoma, and not its own entity, until better characterized.

## 1. Introduction

Infection with human papillomavirus (HPV) is now an established risk factor for many head and neck carcinomas (HNSCCs), including tonsillar and base-of-tongue squamous cell carcinomas (TSCC and BOTSCC, respectively), and a subset of sinonasal nonkeratinizing squamous cell carcinomas (NKSCCs) [1]. However, while the favourable prognostic role of HPV in TSCC and BOTSCC is well established [2,3], the prognostic role of HPV in other HNSCCs, including sinonasal NKSCC, is not yet clearly defined [1].

Adenoid cystic carcinomas (AdCCs) are rare salivary gland malignancies, with a varying clinical outcome [4]. Their risk factors and aetiological agents are mainly unknown, but genetic studies have identified the presence of the fusion transcript of MYB-NFIB in a majority of these tumours [4,5]. However, recent studies have demonstrated the presence of oncogenic viruses in salivary gland tumours in general, and both HPV and human polyomaviruses (HPyVs) have been described in AdCC [6,7,8,9,10]. Irrespectively, the prevalence of HPV and HPyV in AdCC of the head and neck has only been examined in a limited number of studies, and although most cases have been reported as virus-negative, HPV and/or HPyV could still potentially play a role in a subset of AdCCs [7,8,10,11,12,13,14].

In 2013, however, a new additional HPV-related form of sinonasal carcinoma, now entitled HPV-related multiphenotypic sinonasal carcinoma (HMSC), was presented in a case report by Bishop and colleagues [15]. Since this first case report in 2013, fewer than 100 cases have so far been described in the literature [16]. Notably, despite the aggressive micromorphological appearance of HMSC, the diagnosis is often associated with a rather indolent clinical course. The most common HPV type detected so far in HMSC is HPV 33, but other high-risk HPV types have also been described. Initially, HMSC was first reported to occur exclusively in the sinonasal region, but a recent case report has suggested that HMSC-like tumours may also occur outside this specific region [16,17].

Initially, HMSC was entitled HPV-related carcinoma with adenoid cystic carcinoma-like features due to histomorphological resemblances with AdCC. Nevertheless, despite different histological origins, many HMSC cases may have in the past, before this form was recognized in 2013, been diagnosed as AdCC [18].

The aim of this study is, therefore, two-fold: Primarily, we want to examine the presence of HPV and HPyV in the so far, to our knowledge, largest cohort of patients with AdCC in the head and neck region, to determine a possible aetiological role of these oncogenic viruses. Secondly, in the same cohort, we want to identify and characterize possible patients with HMSC diagnosed in Stockholm 2000–2012, either in, or possibly also outside, the sinonasal region.

## 2. Materials and Methods

### 2.1. Patients and Tumour Samples

In total, 94 patients were identified through the Swedish Cancer Registry (Cancerregistret) as diagnosed with AdCC within the head and neck region (ICD-10: C00.5, C01.9, C04.9, C05.9, C06.9, C07.9, C08.0, C09.9, C11.9, C30.0 and C31.9) between 2000 and 2012 in the County of Stockholm. For the continued analysis, haematoxylin–eosin (HE)-stained slides and formalin-fixed paraffin-embedded (FFPE) tumour samples from these patients were collected, when possible, from the Pathology Dept. at Karolinska University Hospital. Sufficient amount and quality of FFPE tumour material was available in 73 samples from 68/94 patients. More specifically, these were from 66 primary tumours; 1 metastasis and 1 local recurrence where no primary was available; finally, 3 local recurrences and 3 metastases where the primaries were available. The study was conducted according to ethical permissions 99-237, 2005/431-31/4, 2009/1278-31/4, 2012/83-31/2, 2017/1035-31/2 and 2019-05211 from the Ethics Committee at Karolinska Institutet, Stockholm, Sweden.

### 2.2. DNA Extraction and Detection of HPV and HPyV

For every patient case, tumour and adjacent normal control tissue were dissected from 10 μm FFPE tissue sections from the same FFPE tissue block. DNA was extracted from tumour and normal tissue sections using the QIAamp DNA FFPE Tissue Kit (Qiagen, Hilden, Germany) following the manufacturer’s instructions and as described previously [19]. Presence of HPV DNA was detected, following a PCR amplification (40 cycles), using a multiplex bead-based assay for 27 different HPV types (HPV 6, 11, 16, 18, 26, 30, 31, 33, 35, 39, 42, 43, 44, 45, 51, 52, 53, 56, 58, 59, 66, 67, 68, 69, 70, 73 and 82), including all high-risk HPV types, and β-globin was included as a positive control for the presence of DNA as described previously [20].

For HPyV DNA analysis, a similar bead-based multiplex assay was used as previously described (3). This assay detects the ST and VP1 regions of 10 HPyVs; BKPyV, JCPyV, KIPyV, WUPyV, MCPyV, TSPyV, HPyV6, HPyV7, HPyV9 and HPyV10, as well as the primate viruses SV40 and LPyV, as described previously [21,22]. β-globin was used as a positive control for the presence of amplifiable human DNA as described previously [23]. The output was assayed as median fluorescent index (MFI) and an MFI value of more than 2 × background + 300 was regarded as a positive value. For MCPyV, this value corresponded to approximately 5 genomes, and values below this were considered of no significance [14].

### 2.3. Immunohistochemistry

Tumour sections were deparaffinized using EZ-prep (Ventana Medical Systems^®^, Tucson, AZ, USA), and immunohistochemistry was performed using the Ventana Benchmark Ultra platform (Ventana Medical Systems). Primary antibodies were directed against p63 (p63 ready to use; Roche 4A4), CD117 (CD117 ready to use; Roche EP10), S100 (S100 ready to use; Roche 4C4.9) and p16 (p16 ready to use; Roche E6H3). Overexpression of p16 was defined as a strong nuclear and cytoplasmatic staining in ≥70% tumour cells, as described elsewhere [24].

### 2.4. Fusion Transcript Analysis

Total RNA was extracted from the FFPE material using the Maxwell^®^ 16 MX3031 instrument with Maxwell^®^ 16 FFPE LEV RNA Purification Kit from Promega (Fitchburg, WI, USA). The quality and quantity of the RNA were estimated through Nanodrop technology (Nanodrop technologies, Wilmington, DE, USA). cDNA was, subsequently, synthesized using the High-Capacity cDNA reverse transcription Kit (Applied Biosystems, Foster City, CA, USA) and RT-PCR was performed in duplicate for targeting the fusion gene transcripts corresponding to MYB-NFIB variants on the LightCycler^®^ 480 II instrument (Roche Diagnostics), according to Fehr et al. [25]. Samples from known AdCC (MYB-NFIB fusion-positive) patients were used as positive controls and the reference gene HPRT1 was included to correct as a positive control.

## 3. Results

### 3.1. Presence of HPV and HPyV DNA in Patients Diagnosed as AdCC

Altogether, 73 tumour samples and corresponding adjacent normal tissues from 68 patients diagnosed with AdCC within the head and neck region were analysed for the presence of HPV and HPyV DNA. The patients and their tumour characteristics are summarized in Table 1. No tumour samples were HPyV-positive, while 3/66 primaries and 1 local corresponding relapse were positive for HPV16 or 33 DNA, as depicted in Table 1; further details are described below.

### 3.2. Patients with HPV-Positive Tumours Diagnosed as AdCC

In patients with HPV DNA-positive tumours, of which one had a sinonasal and two had a tonsillar primary location, patient and tumour characteristics were examined and scrutinized in more detail. Furthermore, to ultimately be defined as HPV-positive, here, we also required the presence of p16 overexpression (see below), since the presence of HPV DNA together with p16 overexpression has earlier been shown to be very close to disclosing the presence of HPV RNA [26].

In all cases, a nonkeratinizing squamous cell carcinoma, a basaloid squamous cell carcinoma or a solid variant of an AdCC were considered as differential diagnoses by the pathology reports. However, the overall conclusion at the time of diagnosis, considering both the histomorphology and immunohistochemistry, was that these tumours represented solid variants of AdCC.

**Figure 1:**
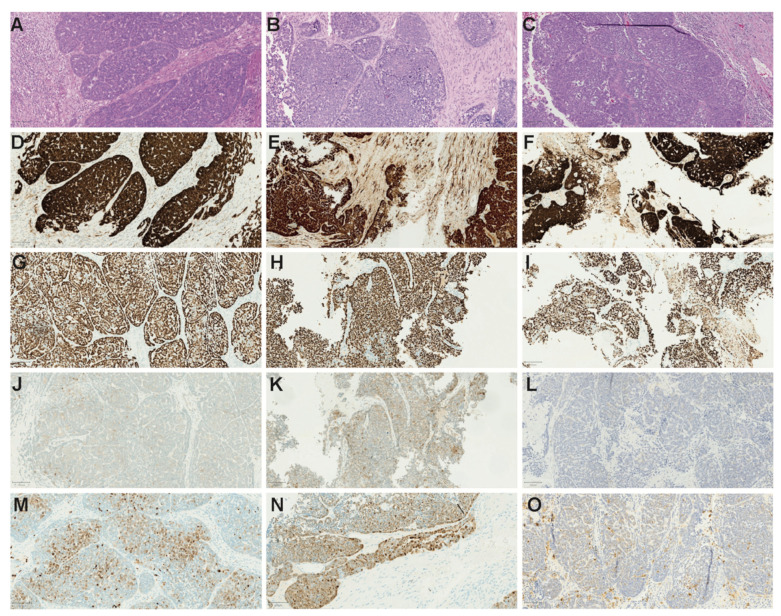
Tumour sections from patients with HPV DNA-positive tumours that were initially diagnosed as adenoid cystic carcinoma stained with haematoxylin–eosin (HE) and immunohistochemical markers. (**A**–**C**) HE. (**D**–**F**) p16. (**G**–**I**) p63. (**J**–**L**) CD117. (**M**–**O**) S100. All tumours showed mainly solid basaloid features, growing in sheets and lobular formations with areas resembling adenoid cystic carcinoma with tubular and cribriform formations. Squamous and myoepithelial differentiation was supported by p63 and S100 staining, with a mixed staining pattern. Moreover, a focal CD117 staining was observed. In addition, all tumours showed overexpression of p16. The micromorphology and immunohistochemistry were similar with findings reported elsewhere [27].

After reviewing the histomorphology, all three primary tumours showed a similar morphology. All were basaloid by their appearance, with small blue basaloid cells with a mainly solid growth pattern and focal areas with tubular- and/or cribriform-like areas. Focally, a tendency to nuclear palisading was observed in all three tumours. In addition, necrotic areas, as well as numerous apoptotic and mitotic bodies, were observed. No clear associated dysplastic squamous epithelium was noticed (Figure 1).

According to the immunohistochemistry, all tumour samples overexpressed p16 (*n* = 4) and were largely diffusely p63-positive. Focal minimal areas with CD117 were also observed in all three primary samples, as indicated in Figure 1. All cases showed a mixed S100 positivity. No MYB-NFIB fusion transcripts associated with AdCC were detected in any of the samples (Table 2). All adjacent normal tissue was HPV DNA-negative when tested by the bead-based multiplex assay.

All three patients with HPV DNA and p16-overexpressing tumours, defined as HPV-positive tumours, presented with early-stage disease upon diagnosis (Table 2); the treatment and clinical outcome, however, varied, as described below:

The first patient, a female (Patient 1, Table 2) with a large paranasal neoplasm, with no disease of the neck detected by diagnostic computer tomography (CT), was treated with conventional radiotherapy (RT) 2 Gy/fraction up to 50 Gy and concomitant Paklitaxel 60 mg/m^2^ once weekly for 5 weeks. A residual tumour was observed after initial treatment and the patient underwent radical surgery and was, subsequently, considered tumour-free until her last regular check-up 73 months later.

The second patient, a male (Patient 2, Table 2) with a tonsillar neoplasm, with no disease of the neck detected by diagnostic computer tomography (CT), was initially treated as an HPV-positive SCC of the tonsil with conventional RT (2 Gy/fraction up to 68 Gy) and considered in remission after initial treatment. However, the patient presented a local recurrence 5 months later, when the diagnosis was also re-assessed and presented as AdCC. He had two extensive surgeries with insufficient margins and signs of residual disease. Moreover, during the first surgery, an ipsilateral neck dissection was performed, where 15 tumour-free lymph nodes were excised. Additionally, after the second surgery, the patient underwent interstitial radiotherapy using the PDR technique, 0.74 Gy/fraction up to 60 Gy with curative intent, but was never considered disease-free after initial recurrence. Palliative chemotherapy (Carboplatin and Paklitaxel) was given, and the patient passed two years after primary diagnosis.

The third patient, a female (Patient 3, Table 2) with a tonsillar mass, with no disease of the neck detected by diagnostic CT, was treated with a radical tonsillectomy, followed by conventional RT 2 Gy/fraction up to 68 Gy and concomitant Cisplatin 30 mg/m^2^ once weekly for 7 weeks. The patient showed no evidence of disease at the last check-up, 90 months after initial diagnosis.

## 4. Discussion

In this large cohort of patients diagnosed with AdCC, HPV and HPyV were shown not to play a major aetiological role. However, we also identified three cases, both within and outside the sinonasal region, that, according to today’s published criteria, could have been diagnosed as the new emerging entity HMSC. Notably, however, the similarities in morphology between these three tumours and basaloid SCC suggest that a better characterization of HMSC may be warranted before HMSC can be justified as its own entity.

Few studies have examined the role of HPV in AdCC, and while some studies, including one study from our group on 13 AdCC, did not show any association between HPV and AdCC [11,12,13], a handful of smaller studies have identified the presence of HPV in a subset of AdCCs.

In this study, however, 3/66 patients that were initially diagnosed with AdCC harboured HPV in their primary tumours but not in their adjacent normal tissue. Nevertheless, after rereviewing the histomorphology, the diagnosis of a squamous cell carcinoma (SCC) with basaloid features, rather than an AdCC, was more likely in these three HPV DNA-positive cases. In fact, it is well recognized that a solid variant of an AdCC in many cases may be hard to distinguish from a basaloid SCC [28] and, therefore, it is important that cases should be morphologically re-evaluated in registry-based studies. Numerous previous studies have, namely, demonstrated a correlation between HPV and basaloid variants of SCC in the head and neck region, as well in the anogenital region [29,30,31]. Thus, it is possible that at least some of the earlier reported HPV-positive cases in previous studies may have represented a basaloid SCC instead of an AdCC, especially if the diagnosis was set before additional molecular and immunohistochemical analyses were introduced and made available in clinical routine. Consequently, in our opinion, should HPV play a role in AdCC, its role would be minor, if present at all.

In similar studies on the occurrence of HPV in AdCC, very few have examined the presence of HPyV in AdCC. In a publication by Hämetoja et al., the presence of two HPyVs (JCPyV and BKPyV) as well as SV40 was analysed in 68 AdCC, and there, 10.3% of the tumours were described to harbour JCPyV in very low copy numbers [10]. Likewise, in a previous study from our group, the presence of 10 different HPyVs was analysed in 99 parotid salivary gland carcinomas, including 12 different histological subtypes. Interestingly, in that report, 9% of all AdCCs harboured the human Merkel cell polyomavirus (HPyV); however, notably, there was no over-representation of any histological subtype with regard to HPyV infection, suggesting a nonspecific role of HPyV infection in these tumours [14]. The data presented here were, therefore, analogous with our previous observations, indicating that the role of HPyV in AdCC should be minor.

Notably, here, we identified three patient cases, one sinonasal and two tonsillar primary tumours with a molecular and a micromorphological profile that fit within the new emerging entity of HMSC, with the exception that two of them were situated outside the sinonasal region. However, in a recent meta-analysis, including all published cases with HMSC so far, two other patients were identified as having HMSC-like tumours outside the sinonasal region [16]. Here, we added another two cases with a profile similar to HMSC, but also diagnosed outside the sinonasal region.

In line with previous reports [27] about this emerging tumour type [16], HPV type 33 dominated with two positive cases, followed by HPV 16 (one positive case). Nevertheless, it is important to remember that both HPV 33 and HPV 16 sort under the same specie (species 9), together with other common types associated with malignant neoplasms in the head and neck region, as well as anogenital malignant tumours [32,33,34].

In the last WHO Classification of Head and Neck Tumours from 2017, HMSC was introduced, but was not considered as its own entity due to too few reported cases in the literature, variable histology with focal overlap with nonkeratinizing SCCs and unknown prognostic significance [35]. Due to a possible similar micromorphological overlap with a basaloid SCC, indicated here as well as in previous studies [28], the occurrence of this morphological pattern outside the sinonasal region [16,17] and unclear clinical significance [16], we suggest that the HMSC, before it is more thoroughly characterized, could possibly be considered a histological subgroup of basaloid SCC.

In analogy with this possibility, a study of basaloid SCCs of the anus is worth mentioning [34]. In that study, 27 HPV-associated basaloid SCCs of the anus were classified into four histological categories, of which one was termed an “adenoid cystic carcinoma-like” basaloid SCC, and had features very similar to HMSC [34]. Nevertheless, basaloid SCC has historically been considered as a rare tumour entity within the sinonasal region, and when described often associated with a worse clinical outcome [36,37]. However, in recent years, there has been a dramatical increase in HPV-associated neoplasms within the head and neck region, especially of the tonsils, which, nowadays, usually present with a basaloid micromorphology and a favourable clinical outcome [38]. One could, therefore, speculate that a similar shift in micromorphology and prognosis also may have occurred in HPV-associated tumours within the sinonasal region.

We acknowledge limitations in this study. This was a retrospective registry-based study. Patients were identified through a national-based cancer registry and identified patients’ pathology and clinical reports were reviewed. Only tumours from the three patients with HPV-positive neoplasms were re-evaluated micromorphologically and with molecular and immunohistochemical analysis. Unfortunately, histological and immunohistology slides were not available for all patients, and it was, therefore, possible that some virus-negative tumours could also have been misclassified as AdCC.

In summary, we suggest that the role of HPV and HPyV is minor, if present at all, in AdCC. We also suggest that HMSC could be considered as a basaloid variant of squamous cell carcinoma, and not as its own entity, since a need for further characterization was indicated.

## 5. Conclusions

To conclude, we found that the role of HPV and HPyV is minor, if present at all, in AdCC. In addition, we indicated that HMSC could be considered as a basaloid variant of squamous cell carcinoma, and not as its own entity.

## Figures and Tables

**Table 1:** Patients initially diagnosed with adenoid cystic carcinoma (AdCC), with patient and tumour characteristics.

Characteristics		N (%)
Total		68 (100)
Gender	Female	44 (65)
	Male	24 (35)
Age	Mean	57
	Median	60
	Range	13–88
Tumour localization	Gl Submandibularis	22 (32)
	Gl Parotidea	20 (30)
	Oral cavity	11 (16)
	Nasal and paranasal sinuses	7 (10)
	Base of tongue/Tonsil	5 (7)
	Lip	2 (3)
	Nasopharynx	1 (2)
HPV DNA	Positive	3 (4)
	Negative	65 (96)
HPyV DNA	Positive	0 (0)
	Negative	68 (100)

**Table 2:** Patients initially diagnosed with adenoid cystic carcinoma (AdCC) and their characteristics.

	Patient 1	Patient 2	Patient 3
Tumour localization	Paranasal sinus	Tonsil	Tonsil
Age ^1^	76	63	67
TNM-8	Stage II	Stage II	Stage II
Treatment	CRT + Surgery	RT	Surgery + CRT
Survival	NED	DOD	NED
HPV type	HPV 33	HPV 33	HPV 16
MYB-NFIB fusion transcript	No	No	No

^1^ Age in years. Abbreviations: CRT—chemoradiotherapy; R—radiotherapy; NED—no evidence of disease; DOD—dead of disease.

## Data Availability

Due to privacy concerns, deidentified data can be made available upon reasonable request to the corresponding author.
